# Identification of key genes and molecular mechanisms associated with low egg production of broiler breeder hens in ad libitum

**DOI:** 10.1186/s12864-019-5801-3

**Published:** 2019-05-22

**Authors:** Zehui Wei, Pengcheng Li, Sijia Huang, Purevsuren Lkhagvagarav, Mengqi Zhu, Chuanyu Liang, Cunling Jia

**Affiliations:** College of Animal Science and Technology, Northwest A&F University, 22# Xinong Road, Yangling, Shaanxi China

**Keywords:** Transcriptome, *Gallus gallus*, Ad libitum, Restricted feeding, Liver, Adipose, Ovary

## Abstract

**Background:**

Overfeeding reduces laying performance in broiler breeder hens, which is associated with obesity, hepatic steatosis and systemic inflammation. To unravel the underlying mechanisms governing the effect of feeding regimes on energy metabolism and egg production, a transcriptomics approach was carried out for screening differentially expressed genes (DEGs) in ovary, liver and adipose tissues of broiler chickens under ad libitum and restricted feeding.

**Results:**

It showed that 289, 388 and 204 DEGs were identified in the adipose, liver and ovary, respectively. These DEGs were significantly enriched in phagosome pathway, lipid transport, activity and nutrient reservoir activity in ovary; steroid hormone biosynthesis and metabolism of xenobiotics by cytochrome P450 pathways in adipose tissue; and the metabolic pathways, peroxisome proliferator-activated receptor (PPAR) and Jak-STAT signaling pathway in liver. Estrogen receptor 1, identified as one of important hubs by constructing PPI network, was up-regulated in ad libitum group, which would make more apolipoproteins be transferred to ovary.

**Conclusions:**

High expression of *VTGs*, *APOB*, *CYBB* and *CTSS* in ovary would induce excess lipid deposit, oxidative stress and potential damage to ovulation. Our results contribute to understanding effects of feeding regimes on metabolic regulation during egg production of broiler breeder hens and also provide new evidence of metabolic regulation from integrated multi-tissue processes.

**Electronic supplementary material:**

The online version of this article (10.1186/s12864-019-5801-3) contains supplementary material, which is available to authorized users.

## Background

Due to genetic selection, commercial broiler chickens (*Gallus Gallus domesticus*) are capable of growing rapidly and yielding meat efficiently [1]. However, the reproductive performance will decline, when broiler breeder hens are feed ad libitum, which is regarded as the result of overeating and also accompanied obesity and fatty livers [2–4]. Restricting feed of broiler hens to about 50 to 60% of full-feeding is a usual practice to prevent obesity, reduce metabolic disorder and increase egg production [5–8]. However, the consequently negative impact on poultry welfare occurs due to prolonged starvation of broiler breeder hens [9]. Thus, it is essential to uncover the mechanism of ad libitum inducing the ovulation problem (eg. defective or non-settable eggs). As we all known, many organs are involved in the process of egg formation, which is a costly process both in energy and nutrient [10]. Among these organs, the ovary is the most important tissue because it is the place of the ovum (i.e., the yolk) development and maturity [11, 12]. Yolk formation depends on lipogenesis and the export of lipid [13–15]. Lipids is primarily de novo synthesized in liver, which are then transported as triglycerides (TGs) to adipose tissues for storage and release or to ovary tissue for the developing oocyte [16, 17]. However, ad libitum feeding broiler hens led to increase lipid synthesis in the liver and change lipoprotein metabolism in whole body that affect yolk formation, and then induced multiple ovulations similar to women with polycystic ovary syndrome, which is related to dysregulate lipid metabolism [10, 14, 18]. In addition, there was higher body fat for broiler breed hen fed ad libitum than that of restricted feeding at sexual maturity, and the abdominal fat weight of ad libitum feeding hens increase at least 50% [19]. Adipose tissue was also regarded as an endocrine organ which can not only secrete multiple hormones but also influence various hormones synthesized from other organs [20, 21]. It was not clearly what role adipose tissue plays in egg production [22, 23]. There were some reports about the effect of ad libitum vs. restricted feeding on the egg production, metabolic hormones, welfare, enzyme and some genes expression in broiler breeders [9, 16, 24, 25]. However, the molecular mechanism regulating these differences by ad libitum vs. restricted has not been completely elucidated.

The purpose of this research was to investigate the genes and their regulations to the nutrition-dependent reproductive ability of broiler breeders from the whole transcriptome level. In this study, expression profiles of ovary, hepatic and adipose tissue were investigated between ad libitum and restricted feeding broiler hens using RNA-seq technology. The results described herein will provide a significant advance in our knowledge about the interrelation of feed intake, lipid metabolism and ovarian function in broiler hens.

## Results

### Gene expression profiles

We systematically analysed sequence data from 18 samples, which included 3 tissues (adipose, liver and ovary) from 6 hens (3 hens from ad libitum group and 3 hens from restricted feeding group). An average of 367.3 million raw reads were detected in 6 samples from each tissue. After filtration, approximately 81% clean reads in each sample were mapped to the Gallus_gallus-5.0 genome, and they were used for further gene expression analysis. There were about 80% reads uniquely and 1% multiple aligned to genome in each sample (Table 1). On average, expression of 12,382, 13,157and 10,651 genes from adipose, ovary and liver tissues were detected respectively, setting the threshold for read counts of each gene above 1 count per million in each sample at least one treatment group. All these expressed genes were used for differential expression analysis.

**Table 1:** RNA-seq reads and mapping rate of different tissues from hens under ad libitum and restricted feeding

Sample ID	Raw reads	Clean reads	Clean bases (G)	Q20 (%)	Multiple mapped	Uniquely mapped	Mapping rate (%)
L1724_R	64,392,818	61,090,010	7.64	95.03	590,134	51,109,302	84.63
L6709_R	59,658,748	56,872,116	7.11	95.28	721,126	48,296,701	86.19
L6710_R	61,714,914	58,355,650	7.29	95.08	609,771	48,887,646	84.82
F1724_R	54,087,908	50,706,756	6.34	94.55	573,191	39,236,404	78.51
F6709_R	59,471,818	55,779,296	6.97	94.61	679,048	43,773,708	79.69
F6710_R	61,929,858	57,824,398	7.23	94.63	657,150	44,024,598	77.27
O1724_R	62,493,328	58,686,194	7.34	94.47	727,917	45,868,843	79.24
O6709_R	61,744,970	58,780,766	7.35	94.99	706,095	48,277,775	83.33
O6710_R	62,257,620	58,644,500	7.33	94.78	699,080	46,991,269	81.32
L1721_ad	58,134,934	55,036,848	6.88	95.02	657,817	45,941,840	84.67
L1725_ad	52,149,722	49,683,928	6.21	95.07	530,491	42,807,229	87.23
L1723_ad	52,895,016	50,549,914	6.32	95.31	579,622	43,351,355	86.91
F1721_ad	74,115,894	69,624,988	8.7	94.87	842,600	54,239,290	79.11
F1725_ad	65,772,032	61,471,800	7.68	94.76	603,536	45,625,211	75.2
F1723_ad	63,539,320	58,562,688	7.32	94.22	748,009	43,141,371	74.94
O1721_ad	62,882,770	59,623,398	7.45	94.96	723,632	48,555,048	82.65
O1725_ad	58,060,322	54,925,226	6.87	94.87	645,717	43,622,886	80.6
O1723_ad	66,648,496	63,031,132	7.88	94.86	781,846	49,771,569	80.2

**Note,** Letter L, F and O represent the sample from liver, adipose and ovary, respectively. Letter R and ad represent restricted feeding group and ad libitum feeding group. The numbers between letters represent the sample number.

### Differentially expressed genes

**Fig. 1:**
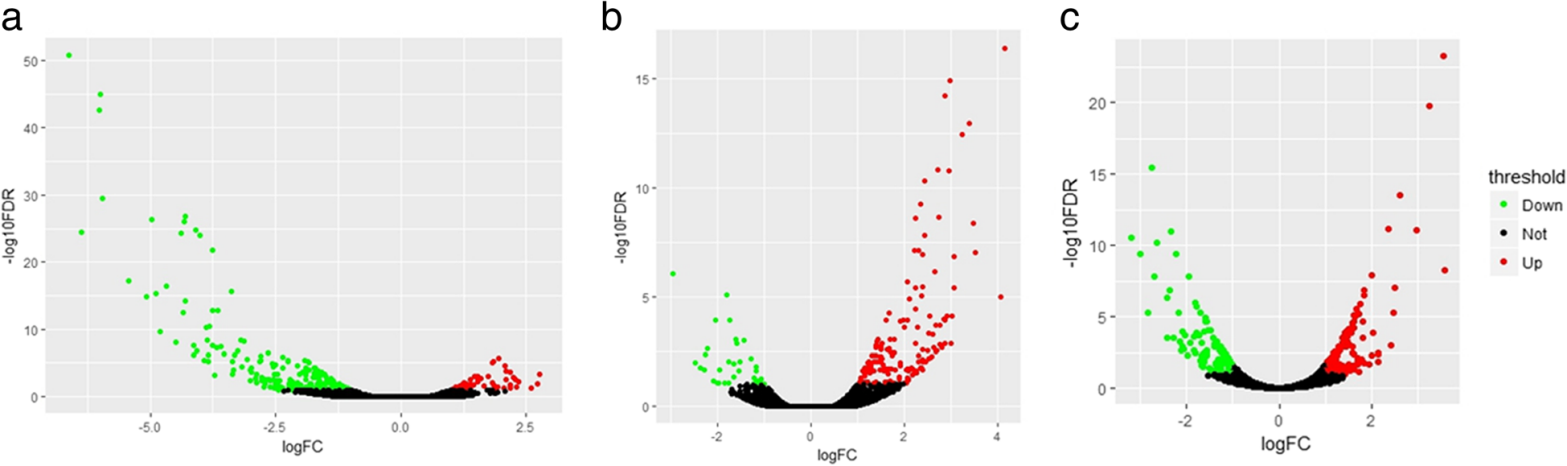
Volcano plot displaying differentially expressed genes between ad libitum and restricted feeding group in three tissues. (a) Adipose tissue, (b) Ovary, (c) Liver. Significantly up-regulated genes were marked by red (Up) (FDR < 0.1); significantly down-regulated genes were marked by green (Down) (FDR < 0.1); insignificantly expressed genes were marked by black (Not)

Differential expression genes were analysed between ad libitum vs restricted feeding group in 3 tissues (FDR < 0.1). The numbers of DEGs were 289 (66 upregulated and 223 downregulated) in the adipose tissue, 388 (240 upregulated and 148 downregulated) in the liver, 204 (170 upregulated and 34 downregulated) in the ovary (Fig. 1, Additional file 2: Table S2). Based on the expression level TPM ≥ 500, the genes are defined as tissue specifically expressed genes (TSEG). There were 356, 499 and 1815 TSEG in the liver, adipose and ovary of ad libitum group (Additional file 3: Table S3). Similarly, there were 418, 345 and 1994 TSEG in the liver, adipose and ovary of restricted feeding group (Additional file 3: Table S3), respectively.

### Gene ontology and functional annotation of DEGs

**Fig. 2:**
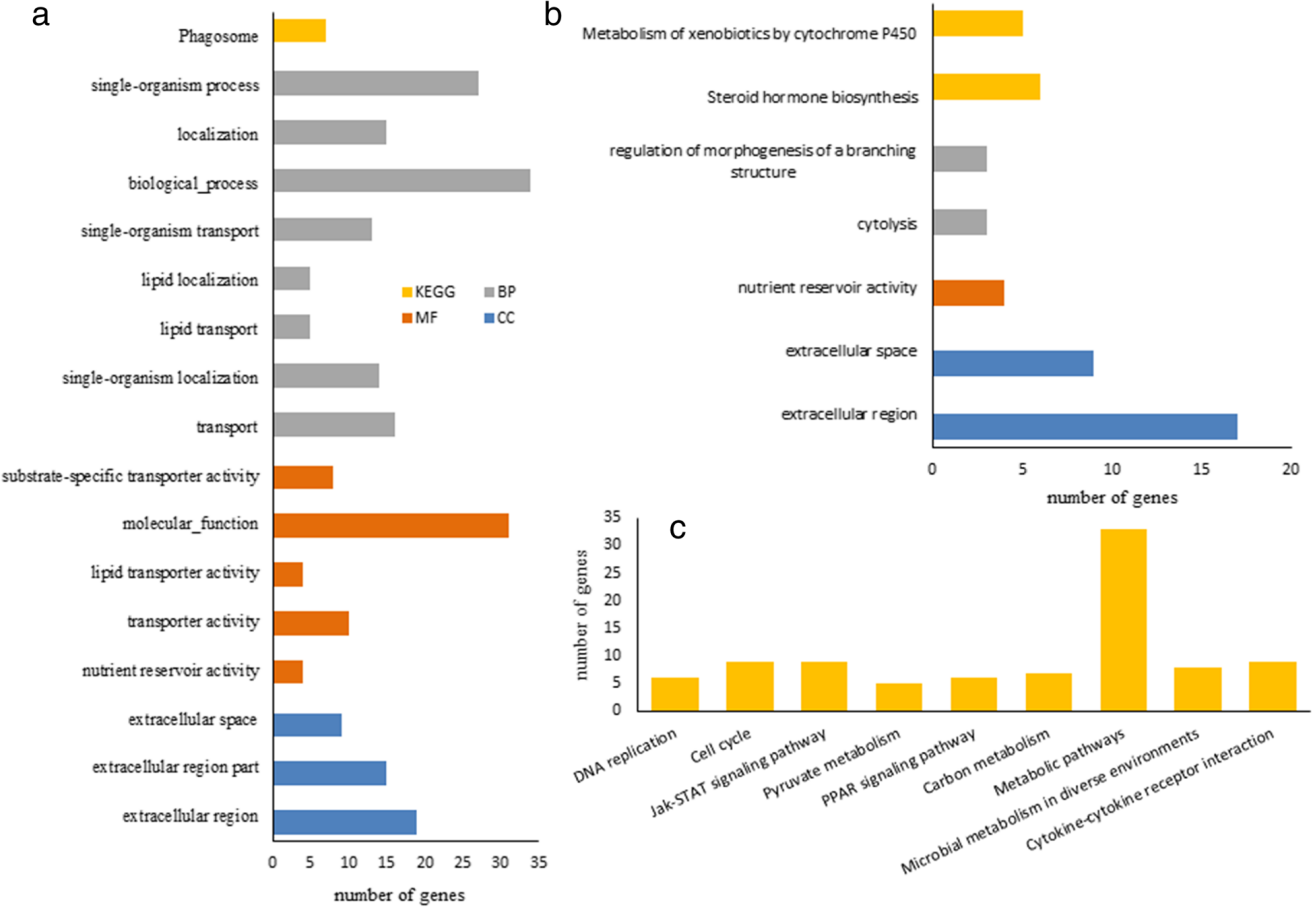
GO analysis and KEGG pathway output of DEGs between ad libitum and restricted feeding group. (a) Ovary, (b) Adipose tissue, (c) Liver. BP, Biological Process; CC, Cellular Component; MF, Molecular Function

Based on the DEGs annotation, enrichment analysis of each tissue from biological process GO terms and KEGG pathway were summarized in Fig. 2 and Additional file 4: Table S4, setting the threshold at FDR < 0.05. DEGs were overrepresented in 5 GO terms and 2 KEGG pathways for the adipose tissue, 16 GO terms and 1 KEGG pathways for ovary, and only 9 KEGG pathways for liver by STRING. KEGG pathway analysis showed that main enrichment pathways were “steroid hormone biosynthesis” and “metabolism of xenobiotics by cytochrome P450” in adipose tissue, “Phagosome” in ovary. In liver, the pathways involved in more genes are Jak-STAT signaling pathway, metabolic pathway, cell cycle and cytokine-cytokine receptor interaction.

### Verification of RNA-seq experiment

**Fig. 3:**
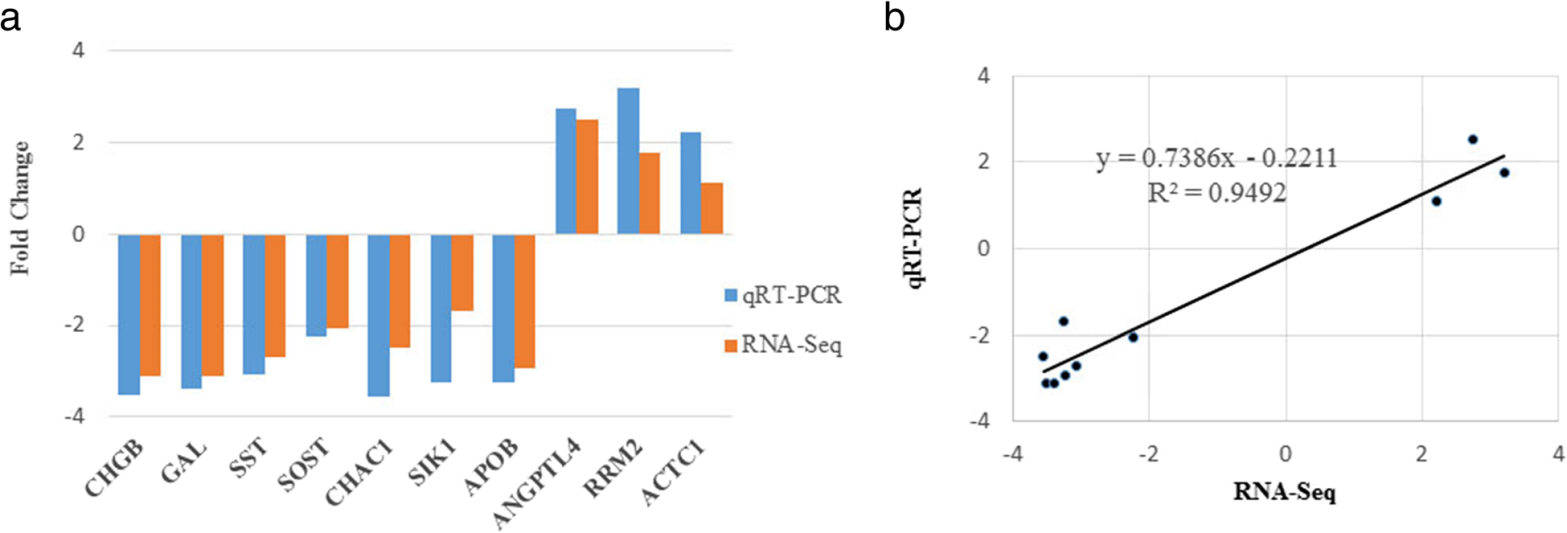
Comparison and correlation analysis of differentially expressed genes between qRT-PCR and RNA-seq. (a) Comparison of differentially expressed genes level from qRT-PCR and RNA-seq. (b) The correlation of differentially expressed genes between qRT-PCR and RNA-seq. *CHGB*, chromogranin B; *APOB*, apolipoprotein B; *SOST*, sclerostin; *GAL*, galanin and GMAP; *SST*, somatostatin; *ACTC1*, actin, alpha, cardiac muscle 1; *CHAC1*, ChaC glutathione specific gamma-glutamylcyclotransferase 1; *SIK1*, salt inducible kinase 1; *ANGPTL4*, angiopoietin like 4; *RRM2*, ribonucleotide reductase regulatory subunit M2. The gene expression was normalized as fold change using *18S ribosomal RNA* (*n* = 3 per group)

The mRNA level of DEG obtained by RNA-seq from ovary, liver and adipose tissues between ad libitum vs restricted feed groups were further examined by qRT-PCR. Figure 3 showed the expression levels of ten genes randomly selected from DEG. For the RNA-seq analysis, the expression of *CHGB, GAL, SST, APOB, SOST, CHAC1* and *SIK1* were down-regulated in ad libitum group by one and a half to three-folds, and the expression of *ANGPTL4, RRM2* and *ACTC1* were up-regulated in ad libitum group by two to four-folds (Fig. 3a). Results of qRT-PCR showed the expressions of these genes were consistent with the results of RNA-Seq analysis, and high correlation was found by comparing results of two methods (Fig. 3b). Therefore, the qRT-PCR data verified that results from RNA-Seq were reliable.

### Protein/protein interaction network of DEGs

**Fig. 4:**
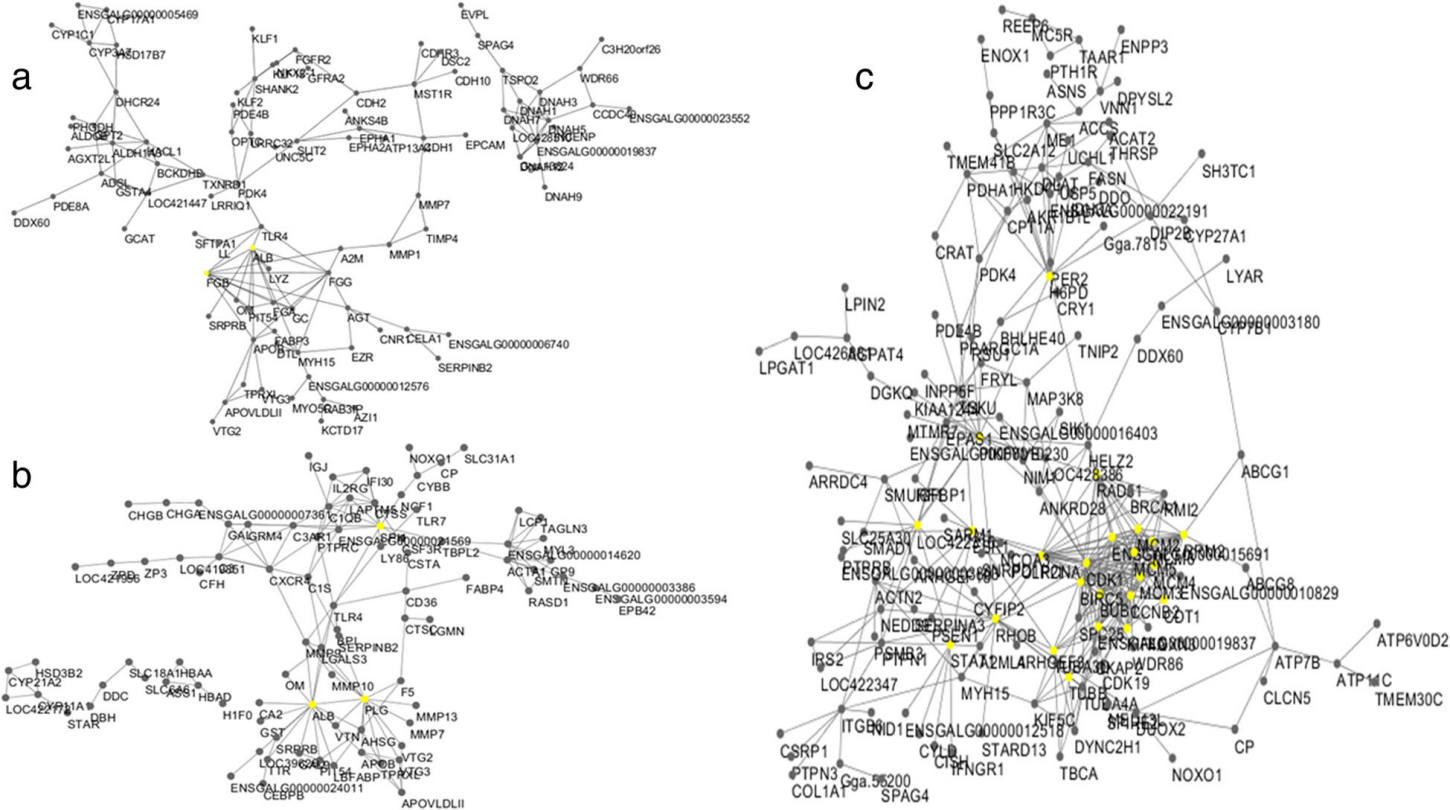
PPI network for DEGs in adipose, ovary and liver tissues. (a) Adipose; (b) Ovary; (c) Liver. In the network, the difference of interaction degree is shown by the color. Yellow dot indicates the node has interaction degree ≥10 considered as hub genes. Black dot indicates the node is differential expression gene. PPI, protein/protein interaction; DEGs, differentially expressed genes

Cytoscape software was employed to visualize PPI networks of DEGs (Fig. 4). It is composed of 123 nodes and 159 edges in adipose, 172 nodes and 373 edges in liver and 100 nodes and 153 edges in ovary. In the figure, node represents DEG and edge represents PPI of two DEGs. There were 2 (fibrinogen beta chain (*FGB*), albumin (*ALB*)) and 3 genes (*ALB*, Spi-1 proto-oncogene (S*PI1*), plasminogen (*PLG*)) identified as hub genes in adipose and ovary with an interaction degree ≥10, respectively, and 24 hub genes in liver.

## Discussion

Restricted feeding of broiler breeder has been widely used to improve the excessive body weight gain and lower egg production efficiency [5–8]. According to the previously reported, unrestricted feeding broiler breeder hen during the laying period accelerates ovarian follicular maturation, which disrupt reproductive performance or increase non-settable eggs [1, 16, 26]. It has been suggested that the reason for unrestricted feeding impairing on egg production of broiler breeder hens was mainly excessive intake leading to excessive energy intake [10, 27, 28]. Despite its high energy requirements, especially yolk formation depending on the metabolism of lipids, broiler breeder hens is little biologic tolerance for overeating [19]. Thus, it is worthy to investigate the underlying molecular mechanisms governing lipid metabolism on egg production, specifically about the effect of nutritional status [16].

In the current study, we conducted transcriptome analysis of the liver, ovary and adipose tissue samples of broiler breeder hen under ad libitum and restricted feeding from 22 wk. to 30 wk. The aim was to examine the impacts of diet intake (ad libitum vs restricted feeding) on the gene expression in these tissues of broiler breeder hens and uncover the molecular mechanism of egg production affected by feed intake. We identified 289, 388 and 204 DEGs using edgeR in the adipose, liver and ovary tissue (FDR < 0.1), respectively.

STRING analysis in ovary showed that DEGs were enriched to phagosome pathway, lipid transport and location biological process, and the molecular function of lipid transporter activity and nutrient reservoir activity (Additional file 4: Table S4). Phagosome is a key pathway in the tissue remodeling and cell phagocytosis of large particles during inflammation [29]. During ovulation, inflammation stimulate connective tissue remodeling, which induce stimulate ovarian follicle rupture to release the mature oocyte. [10, 30]. In our study, the phagosome pathway included 7 DEGs (*ATP6V0D2, C3, CD36, CTSS, CYBB, NCF1* and *TLR4*), and all these gene were significantly up-regulated in ad libitum group compared to restricted- feeding group (Additional file 2: Table S2). Among these genes, beta polypeptide (*CYBB*) and cathepsin S (*CTSS*) genes took part in the process of reactive oxygen species (ROS) [31, 32]. In vervet, higher mRNA levels of *CYBB* will lead to excess ROS production in corpus luteum, which provoke oxidative stress and potential injury to the corpus luteum cells and subsequently influence ovulation [33]. Conventional opinion is that the liver cells are the primary sites of fatty acids synthesis for chickens and they were transported as triglycerides in the plasma by apolipoproteins to the ovary, the adipose tissue for storage and other specific tissues in response to nutritional or physiological need [16, 17]. We found indeed the top five high expression genes (*VTG2*, *ALB*, *APOB*, *VTG1*, *APOV1*) in liver by TMP analysis (TMP > 40,000) were related to lipid transport in both groups (Additional file 3: Table S3). Moreover, these genes were significantly down-regulated in adipose and significantly up-regulated in ovary tissue in ad libitum group compared to restricted-feeding group. Vitellogenin (VTG), one of major yolk-forming components, has been shown to interact with a 95-kDa protein present in detergent extracts of ovarian membranes [34, 35]. Approximately 7% of yolk lipid and 23% of yolk solids were contributes by VTG in chicken eggs [10]. It is reported that VTGs are also synthesized in ovaries of the zebrafish and maybe transported to the close oocytes more efficiently than those transported in the blood from the liver [36, 37]. Apovitellenin 1 (APOV1) and Apolipoprotein B (APOB), the primary apoproteins of very low density lipoprotein (VLDL) particles, are synthesized in the liver and highly stimulated by estrogens at sexual maturity [38–41]. Their mRNA is extremely abundant in the liver [42]. It is thought that APOV1 is likely present only in egg- producing hens, and privileged accumulation. APOB will damage other apolipoproteins present in the immature hen (apo-AI, C-type derived apolipoproteins) [39]. It was suggested that these up-regulated genes in ad libitum made excess energy being transported to the ovary and increase deposition of egg yolk proteins in the developing oocytes, which leaded to multiple ovulations [5, 28]. It may be the one of reason for broiler breeder hens with poor reproductive performance in ad libitum*..*

In adipose tissue, STRING analysis showed that DEGs were most enrichment in the pathways of “steroid hormone biosynthesis” and “metabolism of xenobiotics by cytochrome P450”, and the molecular function of nutrient reservoir activity (Additional file 4: Table S4). Adipose tissue is one of the major endocrine gland and is considered to be one of the main sites of extra-gonadal synthesis of estrogen [43–45]. We found the DEGs in these two pathways were mainly down-regulated (Additional file 2: Table S2) in ad libitum group compared to restricted-feeding group. The role of hormonal and metabolic signals from adipose tissue needs to be studied farther in connection with reproductive function in poultry.

STRING analysis in liver showed that DEGs were mainly enriched to 9 KEGG pathways, as we all know metabolic pathways, Jak-STAT signaling pathway and PPAR signaling pathway were involved (Additional file 4: Table S4). Furthermore, by constructing PPI network for DEGs from ad libitum vs restricted feeding, 24 hubs genes were found (Fig. 4). Among these, estrogen receptor 1 (*ESR1 or ER-α*) was involved in metabolic pathways. The liver is one of mainly responsible organ to estrogens, and the expression of liver estrogen receptors (ERs) is strictly regulated by the reproductive functions and energy homeostasis [46–48]. In liver, the synthesis of egg yolk proteins is highly dependent on estrogen and mediated by ERs [49]. Synthesized apolipoproteins can be transferred via the blood to the developing oocytes [50]. In our study, the mRNA levels of *ESR1* were up-regulated in ad libitum group compared to restricted feeding group, which may increase the synthesis of proteins precursors such as VTG II and APOV1, so that accelerated ovarian follicular maturation and lead to disrupt reproductive performance or increase non-settable eggs.

## Conclusion

Egg yolk deposit refer to the synthesis, absorb, transport and metabolism of lipid from integrated multi-tissue processes. Ad libitum will induce the excess of egg yolk deposit and production of defective or non-settable eggs in broiler breeder hens. In this study, we identified key DEGs, main biology process and regulating pathway by comparing ad libitum with restricted feeding. High levels of *ESR1* mRNA in liver will make more apolipoproteins be transferred to ovary. High expression of *VTGs*, *APOB*, *CYBB* and *CTSS* in ovary will induce excess lipid deposit, oxidative stress and potential damage ovulation. All these results may reveal the molecular mechanism of low egg production of broiler breeder hens in ad libitum*.* However, additional studies should be investigated on the role of hormonal and metabolic signals from adipose tissue in connection with reproductive function in broiler breeder hens.

## Methods

### Animal management and tissue preparation

A flock of Arbor Acres (AA) broiler breeder chicks were reared according to AA Breeder Management Guide specifications to reach target body weight until 21 wk. of age (http://ap.aviagen.com/brands/arbor-acres/). From 22 wk. of age, forty chicks were divided into two groups and transferred into individual laying cages. One group was fed ad libitum with the ration 11.74 MJ/kg metabolizable energy and 17 g crude protein/100 g feed (ad libitum group), and the other group was fed continually on the above guide (restricted group). Access to water was unrestricted during the rearing period. Hens were fed on a lighting schedule according to recommendation from the breeder company. During the laying period, the lighting schedule was 14 h light: 10 h dark. Until 30 wk. of age, 3 hens randomly selected from each group were anesthetized with isoflurane before necropsy. Liver, abdominal fat and ovarian stromal tissues including small follicles were collected at necropsy. Tissue samples were frozen immediately in liquid nitrogen and stored at − 80 °C for farther transcriptome and qRT-PCR analyses.

### RNA extraction

Total RNA was extracted from 18 samples (including adipose, liver and ovary, 6 individuals of each tissue) by TRIzol LS Reagent (Invitrogen, USA). RNA purity and concentration were evaluated with the NanoPhotometer® spectrophotometer (IMPLEN, CA, USA) and Qubit® RNA Assay Kit in Qubi^t^® 2.0 Flurometer (Life Technologies, CA, USA). The RNA integrity was checked using the RNA Nano 6000 Assay Kit of the Bioanalyzer 2100 system (Agilent Technologies, CA, USA) and all of the RNA Integrity Number were more than 9.0.

### Library preparation and transcriptome sequencing

The cDNA libraries were built using NEBNext® Ultra™ RNA Library Prep Kit for Illumina® (NEB, USA). The Agilent Bioanalyzer 2100 system was used to measure the quality of the libraries from 18 samples. An Illumina® HiSeq 2500 platform at Novogene Bioinformatics Technology Co. Ltd. (Beijing, China) was used to sequence all the libraries and the paired-end reads length is 125 bp.

### Reads quality control, alignment and annotation

We used FastQC (Version 0.11.5) to conduct qualify control of raw reads [51]. We removed adapters and low quality reads. After filtered, reads were mapped to the Gallus_gallus-5.0 reference genome using TopHat v2.1.1. [52] incorporates the bowtie v2.0.6 algorithm [53]. Subsequently, the analyses were conducted within R (Version 3.4.0) using packages GenomicFeatures [54] for computing overlaps, and GenomicAlignments [54] for counting short reads mapped within genomic regions on *Gallus gallus* genome GTF file.

### Identification of differential expression gene

Differentially expressed genes (DEGs) were analysed using the R package edgeR [55]. The trimmed mean of M-value (TMM) method was used for normalizing and the generalized linear model (negative binomial) was used for model fitting. Genes with false discovery rate (FDR) < 0.1 were identified as DEGs. To identify tissue specifically expressed genes, transcripts per million (TPM) was calculated using RSEM software [56]. The genes with TPM ≥ 500 were considered to tissue specifically expressed.

### Bioinformatics function analysis of DEGs

The Ensembl transcript ID was used as the primary identifier for all our analyses. The Gene Ontology (GO) terms and Kyoto Encyclopedia of Genes and Genomes (KEGG) pathways were analysed by the online STRING tools (http://string-db.org/) to classify the function of DEGs (FDR < 0.05).

### Real time PCR confirmation

The quantitative real-time PCR (qRT-PCR) was used to confirm the results of DEG from RNA-seq. Total RNA of liver, ovary and adipose samples were extracted using TRIzol Reagent (Invitrogen, USA), and 1 μg total RNA were reversely transcribed to cDNA using the PrimerScrip™RT reagent Kit (Takara, Japan). The qRT-PCR kit SYBR^Ⓡ^Premix Ex Taq™ II (Tli RNaseH Plus, Takara, Japan) was used to determine expression of 10 mRNA. The qRT-PCR reaction was performed in the ABI 7300 Real-Time PCR system (Applied Biosystems, Foster City, CA). The primer sequences (TSINGKE Biotech, China) are provided in Additional file 1: Table S1. The 15 μl PCR total mixed volume in each well contained 7.5 μl SYBR1 Fast qPCRMix (Takara, Japan), each forward and reverse primer 0.8 μl (10 μmol/L), 0.3 μl ROX Reference Dye, 1 μl cDNA template and the rest was RNase-free H_2_O. The PCR reaction was conducted as follows: 30 s at 95 °C, followed by 40 cycles each for 5 s at 95 °C, and annealing temperature of each primer for 34 s and 30 s at 72 °C. The 2^−ΔΔCT^ method was used to calculate target gene expression, and *18S* was used as an internal control.

### Protein/protein interaction network analysis of DEGs

The STRING database was performed to analyse protein/protein interaction network (PPI) of DEGs. At least one connection with a protein and a medium confidence score (≥0.4) were set as cutoff criteria. The networks in liver, adipose or ovary were built according to the known interaction in chicken by extracted DEGs gene list from the database. Cytoscape software (version 3.4.0) [57] were used to visualize PPI networks. The degree for each gene was calculated by analysing the topological structure of PPI network. The gene with degree ≥10 was considered as the hub genes in the network.

## Additional files

Additional file 1: **Table S1.** Primer sequences and product sizes. (DOC 49 kb)

Additional file 2: **Table S2.** The differentially expressed genes in three tissues between ad libitum and restricted feeding group. (XLSX 3189 kb)

Additional file 3: **Table S3.** The tissue specifically expressed genes (TPM ≥ 500) in three tissues of ad libitum group. (XLSX 215 kb)

Additional file 4: **Table S4.** Gene Ontology (GO) and KEGG pathway of differential expression genes in adipose, ovary and liver between ad libitum and restricted feeding group. (XLSX 14 kb)

## Abbreviations

AA: Arbor Acres
*ACTC1*: Actin, alpha, cardiac muscle 1
*ALB*: Albumin
*ANGPTL4*: Angiopoietin like 4
*APOB*: Apolipoprotein B
APOV1: Apovitellenin 1
BP: Biological Process
CC: Cellular Component
*CHAC1*: ChaC glutathione specific gamma-glutamylcyclotransferase 1
*CHGB*: Chromogranin B
*CTSS*: Cathepsin S
*CYBB*: Beta polypeptide
DEGs: Differentially expressed genes
ERs: Estrogen receptors
*ESR1*: Estrogen receptor 1
FDR: False discovery rate
*FGB*: Fibrinogen beta chain
*GAL*: Galanin and GMAP prepropeptide
GEO: Gene expression omnibus
GO: Gene Ontology
MF: Molecular Function
*PLG*: Plasminogen
PPAR: Peroxisome proliferator-activated receptor
PPI: Protein/protein interaction network
qRT-PCR: quantitative real-time PCR
ROS: Reactive oxygen species
*RRM2*: Ribonucleotide reductase regulatory subunit M2
*SIK1*: Salt inducible kinase 1
*SOST*: Sclerostin
S*PI1*: Spi-1 proto-oncogene
*SST*: Somatostatin
TGs: Triglycerides
TMM: Trimmed mean of M-value
TPM: Transcripts per million
VLDL: Very low density lipoprotein
VTG: Vitellogenin
