# Supplementary material for: Identification of key genes and molecular mechanisms associated with low egg production of broiler breeder hens in ad libitum
Source: BMC Genomics. 2019 May 22;20:408. doi: 10.1186/s12864-019-5801-3 (PMC6532148; doi:10.1186/s12864-019-5801-3)
Supplement: Supplementary file 1 — Table S1. Primer sequences and product sizes. (DOC 49 kb) [file 12864_2019_5801_MOESM1_ESM.doc]

**Table S1. Primer sequences and product sizes**

| Gene symbol | Accession number | Primer sequence | Annealing temperature | Product size (bp) |
| --- | --- | --- | --- | --- |
| SOST | CR353045 | F: GCCTCTGCTAATCTGGAAGTC  R: CCAAGTTGTGTTCACCTGATG | 63.1 | 212 |
| RRM2 | HAEL01008561 | F: CGTGTGCTTCTTCTCCTGGTATGT  R: TCGCCTGCCATGCCAAGTTC | 55.5 | 102 |
| ANGPTL4 | XM_001232283 | F: AGCCACACCAACCAGAGCCATA  R: ATCACCGTCCAGCCACCTTCT | 63.7 | 185 |
| CHAC1 | HAEK01040348 | F: TACATCGCCACGCCGCAGAA  R: ATGAAGTCCACCAGCCGCAG | 67.5 | 131 |
| APOB | DQ630943 | F: CGGCTCTTGCTGCTCTTGCT  R: ACCGCTTCGAGAATCTGCTGTT | 58.4 | 181 |
| SIK1 | AF219232 | F: GGTGCTTCTCTCCTGCCTGTCA  R: TGTTCGGCTGTGCGTGTTGG | 63.1 | 226 |
| ACTC1 | HAEK01014447 | F: TGAGGCTGGTCCATCCATTGTC  R: ATGACGGAGGAGTTCAGGAAGG | 63.1 | 130 |
| CHGB | XM_004935278 | F: AGTCTTGGCTGGCATCTCAC  R: TTGGCTTAGACAGAGCATTGG | 65.5 | 123 |
| GAL | XM_015286791 | F: GAAGGAGCCCGTAGGCAATTC  R: GACTGTGCGGCTGATCTCTAGG | 67.1 | 175 |
| SST | EF593949 | F: GCCGAGCAGGATGAAGTGAGAC  R: GGGAGGACAGGTGGGTTTCAA | 66 | 147 |
| 18S | XR_003078044 | F:TAGTTGGTGGAGCGATTTGTCT  R:CGGACATCTAAGGGCATCACA |  | 169 |

*CHGB*, chromogranin B; *APOB*, apolipoprotein B; *SOST*, sclerostin; *GAL*, galanin and GMAP; *SST*, somatostatin; *ACTC1*, actin, alpha, cardiac muscle 1; *CHAC1*, ChaC glutathione specific gamma-glutamylcyclotransferase 1; *SIK1*, salt inducible kinase 1; *ANGPTL4*, angiopoietin like 4; *RRM2*, ribonucleotide reductase regulatory subunit M2. *18S, 18S ribosomal RNA* was used as an internal control.
